# Inter-rater reliability and aspects of validity of the parent-infant relationship global assessment scale (PIR-GAS)

**DOI:** 10.1186/1753-2000-7-17

**Published:** 2013-05-24

**Authors:** Jörg M Müller, Sandra Achtergarde, Hanna Frantzmann, Kathrin Steinberg, Olena Skorozhenina, Thomas Beyer, Tilman Fürniss, Christian Postert

**Affiliations:** 1 Department of Child and Adolescent Psychiatry, University Hospital Münster, Schmeddingstr. 50, Münster, 48149, Germany; 2 Department of Applied Health Sciences. Study Programme Occupational Therapy, University of Applied Sciences, Universitätsstraße 105, Bochum, D-44789, Alemanya

**Keywords:** *DC:0–3*, *DC:0-3R*, PIR-GAS, Parent-infant relationship global assessment scale, Inter-rater reliability, Observation time

## Abstract

**Background:**

The Parent-Infant Relationship Global Assessment Scale (PIR-GAS) signifies a conceptually relevant development in the multi-axial, developmentally sensitive classification system *DC:0-3R* for preschool children. However, information about the reliability and validity of the PIR-GAS is rare. A review of the available empirical studies suggests that in research, PIR-GAS ratings can be based on a ten-minute videotaped interaction sequence. The qualification of raters may be very heterogeneous across studies.

**Methods:**

To test whether the use of the PIR-GAS still allows for a reliable assessment of the parent-infant relationship, our study compared a PIR-GAS ratings based on a full-information procedure across multiple settings with ratings based on a ten-minute video by two doctoral candidates of medicine. For each mother-child dyad at a family day hospital (N = 48), we obtained two video ratings and one full-information rating at admission to therapy and at discharge. This pre-post design allowed for a replication of our findings across the two measurement points. We focused on the inter-rater reliability between the video coders, as well as between the video and full-information procedure, including mean differences and correlations between the raters. Additionally, we examined aspects of the validity of video and full-information ratings based on their correlation with measures of child and maternal psychopathology.

**Results:**

Our results showed that a ten-minute video and full-information PIR-GAS ratings were not interchangeable. Most results at admission could be replicated by the data obtained at discharge. We concluded that a higher degree of standardization of the assessment procedure should increase the reliability of the PIR-GAS, and a more thorough theoretical foundation of the manual should increase its validity.

## Introduction

The Zero To Three Taskforce [1] published the *Diagnostic Classification of Mental Health and Developmental Disorders of Infancy and Early Childhood (DC:0–3)* in 1994 to address the need for a systematic, developmentally based approach to the classification of mental health and developmental disorders in the first four years of life [1]. Most classification categories contained in the *DSM-IV* and *ICD-10* were derived from psychopathology in adults, adolescents, and school-age children. The *DC:0–3* and the revised *DC:0-3R*^*a*^[2] represent a developmentally sensitive addition to the available classification systems and take key aspects of the relationship between the infant and primary caregiver into account. Therefore, the *DC:0-3/DC:0-3R* may complement, but not replace, existing classification systems [3,4].

Specifically, the *DC:0-3/DC:0-3R* offers the following two measures to assess the quality of the parent-infant relationship: the *Parent-Infant Relationship Global Assessment Scale* (PIR-GAS; [1,2]) and the *Relationship Problems Checklist* (RPCL; [2]). Both measures are directly integrated into the multi-axial scheme (described below). Developing reliable measures to assess relationships and related disorders is an empirical challenge [5]. Beginning with a discussion of the importance of relationship assessment, this paper provides an overview of the application of the PIR-GAS in research studies, reflects the standards of the manual, and describes an empirical study that examined the influence of specific assessment issues on reliability. Finally, potential improvements in the application of the PIR-GAS are suggested.

### The conceptual role of the mother-child relationship in the DC:0-3/DC:0-3R

The *DC:0-3/DC:0-3R* assumes that the relationship between the infant and primary caregiver plays a major role in the development of psychiatric symptoms and the treatment of these symptoms and that it may, in itself, constitute a specific diagnostic entity for the infant and preschool age. Olson and colleagues [6] and Shaw and colleagues [7] demonstrated the interplay of individual and relationship factors in the pathogenesis of early childhood mental illness using a child’s difficult temperament and negativity in the mother-child interaction to predict externalizing disorders. In studies conducted by Minde and Tidmarsh [8] and Keren and colleagues [9], 53% to 73% of a clinical sample fulfilled the DC:0–3 criteria for the diagnosis of a relationship disorder. In a Danish general population sample, this rate was 8.5%, and there was a significant association between having a relationship disorder and the occurrence of hyperactivity/attention deficit disorder, reactive attachment disorder, disorder of conduct and emotions, or regulatory disorders [10]. Thomas and Clark [11] found that disorders of affect were significantly more likely to occur in combination with relationship disorders than disorders of regulation or posttraumatic stress disorder. In summary, disorders in the relationship between young children and their parents seem to be a frequent problem, especially in clinical samples [12]. This issue justifies the inclusion of relationship disorders as an axis in a multi-axial diagnostic system.

### The multi-axial scheme of the DC:0-3/DC:0-3R

The *DC:0-3/DC:0-3R* represents a multi-axial assessment scheme that is comprised of clinical disorders of the early childhood (Axis I) with a relationship classification on Axis II. Medical and developmental disorders (and conditions) are included on Axis III. Axis IV describes psychosocial stressors as potential risk factors, and Axis V, which may also serve as an outcome measure, focuses on emotional and social functioning. This multi-axial diagnostic approach accounts for the classification of disorders and assigns areas of diagnostic assessment. Because the *DC:0-3/DC:0-3R* was intended to complement existing classification systems, such as the *DSM-IV* and *ICD-10*, its structure has great overlap with these systems, despite clear developmental adjustments. One exception in terms of these overlaps is the relationship classification coded on Axis II, which has a novelty character. The associated PIR-GAS is more prominent in the revised version *DC:0-3R*[2] after having been moved from an appendix to the main text. Additionally, the related issue of relationship disorder subtypes (i.e., the classification of a disordered relationship as *overinvolved*, *underinvolved*, *anxious/tense*, *angry/hostile,* or *mixed*) has been transferred to the new *Relationship Problem Checklist* (RPCL, [2]).

### The PIR-GAS

The PIR-GAS allows for a global rating of the quality of a parent-infant (or parent–child) relationship on a numerical scale, with higher scores indicating higher relationship quality. With the revision of the DC:0–3, the PIR-GAS scoring system has also been revised, and the current version (in DC:0-3R) differs in some aspects from the original version. In the empirical literature, we found some results that rely on the original version, while others rely on the revised scoring system. To render different findings comparable, we contrasted the original and revised PIR-GAS scoring system. Additionally, we reviewed current information regarding the psychometric quality of the scale.

#### Original and revised version

The original and revised versions of the PIR-GAS are presented in Table 1[1,2,13]. In the first column, the labels of different ranges of relationship quality are listed. These ranges are described in the manual with a list of criteria that are considered to be typical for a specific quality range (not included in detail in the table). In the second and third columns, the numerical expressions of these ranges are given for the original and revised version, respectively. The fourth column expresses the clinical severity of the ranges of relationship quality. As observed in Table 1, the labels of the ranges of relationship quality and their clinical interpretation are the same in both versions. There are two main differences between the two versions. First, the revised version includes an additional category at the low end of relationship quality, namely, “documented maltreatment”. Second, the revised version starts at “one”, whereas the original scale starts at “ten”. Keeping these differences in mind, Table 1 can be used to transfer PIR-GAS ratings based on the original scoring system into ratings according to the revised scoring system, and vice versa.

**Table 1:** PIR-GAS in DC:0–3 and DC:0-3R; ranges of relationship quality, numerical expression, and clinical interpretation

**Quality of relationship**	**Original (DC:0–3) Score**	**Revised (DC:0-3R) Score**	**Classification according to clinical severity**
Well adapted	90	91–100	Adapted relationship
Adapted	80	81–90	
Perturbed	70	71–80	Disturbed relationship
Significantly perturbed	60	61–70	
Distressed	50	51–60	
Disturbed	40	41–50	
Disordered	30	31–40	Disordered relationship
Severely disordered	20	21–30	
Grossly impaired	10	11–20	
Documented maltreatment*	–	1–10	

*Not included in the original DC:0–3 version.

#### Degree of standardization of the PIR-GAS

The value of any classification or scoring system can be expressed by its reliability and validity, with replicability and precision being key issues [14]. Studies of the reliability and validity of the *DC:0-3/DC:0-3R* are rare [15-18]. Reliability research focuses on independence from the variation of assessment conditions. These assessment conditions are comprised of the setting (e.g., time of observation; free play situation vs. structured task), characteristics of the observer/rater (degree of experience with preschoolers with mental health problems), the rated criteria, and the integration of additional clinical information. As the PIR-GAS is an observational instrument, inter-rater reliability is of primary concern and is a basic precondition for validity. However, a closer look at the PIR-GAS manual reveals that several aspects of standardization have not yet been determined (Table 2). These uncertainties in the manual could make it difficult to produce reliable and comparable ratings.

**Table 2:** PIR-GAS manual excerpts on reliability aspects and authors’ comments

**Manual excerpt **[2]	**Comment by the authors**
#1 “A skilled clinician [who conducts a diagnostic evaluation and formulates an intervention plan] can use the concepts and measures in Axis II to formulate and focus interventions.” (p. 41f)	The qualification of raters does not focus on explicit skills, e.g., specific training, or years of professional experience with children. It remains unclear whether any member of a multi-professional team (including several professional disciplines, such as child and adolescent psychiatrists, nurses, and pedagogical staff) with various levels of clinical experience can provide an equivalent rating quality. Additionally, there is a scientific demand for independent diagnostic information, e.g., by third-party raters.
#2 “In assessing the parent-infant relationship, the clinician should consider multiple aspects of the family dynamic (overall functioning level, level of distress and adaptive flexibility in both the child and the parent; level of conflict and resolution between the child and the parent; effect of quality of the relationship on the child’s developmental progress.” (p. 41f)	The manual describes several global issues, or potential psychometric subdimensions of the PIR-GAS, such as functioning or distress, that are related to family dynamics. It appears that these subdimensions play different roles across the range of relationship quality. The manual does not name distinct observable criteria for these potentially different aspects and does not specify how to document them. Individual child and parental distress, for example, should be separated from the stress that arises from relationship problems. Furthermore, there is no guideline regarding how to weigh and integrate contradictory information.
#3 “The clinician typically completes the scale after multiple clinical evaluations for a referred problem.” (p. 42)	To reliably apply the PIR-GAS, the user needs to know how long, how often, in how many and in what type of situations (alone or with the mother, siblings, or others) the child and primary caregiver should be observed. What is an acceptable minimum to yield reliable ratings? It would be interesting to know whether and how a typical PIR-GAS-observation-situation could be defined.
#4 “Diagnoses of relationship disturbances or disorders are made not only on the basis of observed behavior but also on the basis of the parent’s subjective experience of the child as expressed during a clinical interview and the subjective experience of the child, as expressed in a play interview, for example.” (p. 42)	The authors recommend a clinical integration of data from different sources and an assessment using different methods, including observations performed by a clinician, the usage of retrospective and current information about the mother-child interaction reported by the mother during a clinical interview, and observation of the child by a skilled clinician in a play interview. Again, documentation and weighting of single observations and their integration are not described. Furthermore, the inclusion of all available information into a final PIR-GAS rating, as recommended in the manual, renders the validation of a PIR-GAS rating difficult, as there are no external criteria left.

The *DC:0-3/DC:0-3R* system, and the PIR-GAS scale in particular, represent suggestions from clinicians about its standardized use in clinical practice ([2], p. 11). However, if the *DC:0-3/DC:0-3R* is to be improved by empirical research, each single measure in the classification system will have to meet scientific requirements to improve the *DC:0-3/DC:0-3R* system as a whole. Our comments regarding the requirements of conducting a PIR-GAS judgment bring to fruition new possibilities for researchers who might apply the PIR-GAS measure to attain the goal of a standardized measure. The current flexibility with the use of the PIR-GAS is exemplified in existing literature, which will be shown in the following section.

#### Empirical results on the inter-rater reliability of the PIR-GAS

The vague recommendations in the manual on how to generate a PIR-GAS rating have led to broad variation in research studies. We show four inter-rater reliability studies with different assessment procedures to yield a PIR-GAS rating (Table 3). For each study, Table 3 reports the rater qualification, the sample description, the description of the materials and setting, the classification procedure (re-scoring), the procedures chosen to describe inter-rater reliability, and the observed inter-rater reliability by correlation, mean score differences, and kappa*.*

**Table 3:** Inter-rater reliability of the PIR-GAS: empirical results

**Reference**	**Rater qualification**	**Sample**	**Material and setting**	**Procedure**	**Inter-rater reliability**
[9]	A trained child psychiatrist and a clinical psychologist.	15 clinically referred children who were younger than 36 months.	The material and setting used for the PIR-GAS ratings were not further described.		Inter-rater-agreement of 92% for relationships diagnoses.
[14]	Trained experts with postgraduate certification in child and adolescent psychiatry, clinical experience.	18 children of a normal population (approximately 18 months old). Fifty percent of these children were at-risk. Among these 18 children, there were two cases with a relationship disorder.	The PIR-GAS rating was based on reviewing the case material, which included a ten-minute videotaped interaction situation.	Examination of the test-retest reliability of the PIR-GAS within a time span of 3 to 12 months. Binary outcomes (PIR-GAS <40 and >40) were compared.	The inter-rater agreement was 100% (kappa = 1), and a test-retest reliability of kappa = 1 was reported.
[19]	Two independent and blinded raters. Not further defined.	53 children (29 boys, 24 girls), 20 months old; mothers with low socio-economic status.	10-minute videotaped interactions between the mother and infant that contained a free play session in a laboratory playroom with a standard set of toys.	Ratings included the following dimensions: ‘behavioral quality of the interaction’, ‘affective tone’, ‘psychological involvement’.	Inter-rater reliability was *r* = .83 (statistic not further defined). Mean score differences between the raters were not reported.
[20,21]	A therapist and an independent psychologist.	75 children who were younger than 18 months and whose mothers were worried about them.	Ratings were based on the interaction between the child and the mother during the interview (from which a ten-minute videotape excerpt was used), as well as on the basis of information provided by the mother.	The first rater uses the interview and information by the mother. The second rater rated 20 pre- and post-treatment interviews (10-minute- videotapes).	Intraclass correlations were r = .90 at admission and r = .86 at discharge. Outcome analyses used rater means.

*Comment:* We do not intend to understate the studies cited here, but have chosen them to describe the different procedures that have been used to conduct a PIR-GAS rating. We do recognize that the main intentions of these four studies were not reliability research.

The variability in conducting a PIR-GAS rating beyond reliability studies can be even greater. PIR-GAS ratings can differ largely with respect to the setting and content of clinical material, which may vary from a retrospective clinical chart review [22] over a 10-minute video sequence [19] up to multiple-sessions diagnostics. A second source refer to the qualification of raters, e.g. from social workers [8], trained child psychiatrist [9] to pediatrician [23]. This heterogeneity may exist because in empirical studies, researchers conduct the PIR-GAS rating according to the specific circumstances of the study, whereas clinicians may conduct a PIR-GAS rating according to the conditions and requirements of the clinical setting. These individual conditions and requirements can vary greatly between research and clinical contexts. For example, the PIR-GAS manual states that for a full evaluation of all five axes, the evaluation “requires a minimum of three to five sessions of 45 or more minutes each” ([2], p. 7f). This amount of time may be adequate in a clinical setting, but it is too expensive in a research context. Accordingly, the literature shows that researchers have tried to lower these costs by diverse measures, for example, by limiting the time span for the observation of parent–child interactions or by closely defining the amount of information to be integrated (Table 3). Another possibility for lowering costs is to rely on novice (e.g., student) ratings rather than exclusively seeking expert judgments.

We would like to know whether such an economical version of the PIR-GAS rating is equivalent in reliability and validity to the ‘classical’, more extensive PIR-GAS rating. If this procedure proves sufficiently reliable and valid, several advantages of the ‘economical version’ might be higher comparability among studies and more research activity, as the ‘economical version’ fits scientific needs much better than the ‘classical’ rating procedure. We addressed these questions in our study.

### Aims of the present study

The primary aim of the present study was to determine whether differences in the assessment procedure have an impact on a PIR-GAS rating. Our study design was primarily motivated by the paper of Aoki [19], which implied that a PIR-GAS rating could be based on a 10-minute video interaction sample by ‘blinded coders’. A first investigation between a PIR-GAS ratings based on full clinical information and a 10-minute-excerpt of a clinical interview with the mother was performed by Salomonsson and Sandell [20] who observed a high intra class reliability. However, the PIR-GAS rating of an external rater was based on a interview recording, and the pre-post treatment status was not covered. We consider it therefore still questionable, whether 10-minute video records of a mother-child interaction sequence render PIR-GAS ratings which are comparable to procedures which fulfill all request from the manual. In the first step, we examined two ratings based on a 10-minute unstructured interaction between mother and child to determine if these two ratings were comparable in terms of how they rated the level of relationship quality. This comparison was based on mean differences, thus expressing raters’ severity. In the second step, we examined if the 10-minute ratings were correlated, as this would demonstrate if they assessed the same content, even if they applied different thresholds. In the third step, we examined the central question of whether the 10-minute ratings PIR-GAS ratings were comparable to full information ratings by an expert group observing the mother-child dyad across multiple settings. Again, we considered mean differences and correlations.

Beyond the primary interest of our study, our data allowed for exploring several other interesting research questions. First, our data consisted of two assessment points, specifically at the beginning of treatment (admission) and at the end of treatment (discharge). This aspect of our experimental design allowed us to replicate our findings from admission with the data from discharge. Moreover, our data also included information about external criteria of a mother-child relationship, namely, child and maternal psychopathology [10]. We examined whether the PIR-GAS ratings based on full clinical information or 10-minute-video were correlated with child and maternal psychopathology, as well as identifying which of the ratings showed higher correlations with these external criteria. Overall, the results should provide empirical evidence regarding whether a 10-minute interaction video may deliver PIR-GAS ratings that are comparable to ratings following all recommendation from the manual.

## Method

### Procedure

#### Sample selection

The Child Psychiatric Family Day Hospital in Münster, Germany, treats infants and preschool children with child psychiatric disorders, using a multi-professional team with a special focus on the mother-child relationship. Since 1997, interaction situations between children and their mothers have been videotaped and archived as part of the routine diagnostic process at admission and discharge of treatment (mean duration of treatment was 22 weeks). The diagnostic process at admission was completed within the first three weeks of attendance, and at discharge, the diagnostic assessment was completed within the last three weeks of attendance.

To avoid possible confusion with siblings of the target child in the video, we only selected families that had one child being treated at the hospital. Our sample consisted of 48 mother-child dyads obtained from the video archive at admission and 36 mother-child dyads obtained from the video archive at discharge. For the majority of cases, the following information was provided: a PIR-GAS full-information rating, a Child-Behavior Checklist (CBCL/1.5.5, see below) to assess child psychopathology, and a Symptom Checklist 90-R score (SCL-90-R, see below) to assess parental psychopathology.

#### Sociodemographic description

The sample included 31 boys (64.6%) and 17 girls (35.4%). The mean age of the children was 3.88 years (*SD* = 1.92). The mean age of the mothers (*n* = 46) was 32.60 years (*SD* = 6.27, range 21–46 years). Forty-six sets of parents (92.00%) were married or living in a common law situation, and four sets of parents (8.00%) were separated or divorced. On average, the families had 1.48 children (*SD* = 0.74, range 1–4).

#### Material

*Video tapes.* For each mother-child dyad that had the necessary information mentioned above, the archived videos were checked to provide a 10-minute video sequence of mother-child interaction at admission and discharge. These sequences were distributed randomly over 16 videotapes. Each tape contained 50% of the parent–child interactions at admission and 50% of the interactions at discharge. Each family appeared only once on each tape. The coders rated the interaction blinded to whether the video was recorded at baseline or discharge status.

### Measures

#### PIR-GAS Coders

Two medical doctoral candidates rated the video material. The coders rated the interaction situations independently from each other and were blinded to all other clinical information. To ensure comparable PIR-GAS ratings, the coders were required to thoroughly study the manual and related literature. Moreover, the coders relied on the definitions of the scoring categories, along with behavior anchors provided by the manual. This assessment procedure is further abbreviated by the term ‘video’.

#### PIR-GAS full-information ratings

At admission and discharge, the quality of each parent–child relationship was assessed and rated by a clinical consensus that involved a group of experienced clinicians (each with approximately two years of working experience in the Family Day Hospital). The group included the senior consultant in child and adolescent psychiatry, child psychiatric interns, developmental psychologists, occupational therapists, psychomotor therapists, and specially qualified nurses. Additional clinical observations and descriptions from parents or daycare centers were discussed within the therapeutic team. There were always two people in the team who worked directly with the target child and parent, while the other members contributed additional information. Therefore, we considered the PIR-GAS full-information rating mainly as a conglomerate of two raters’ judgments. This assessment procedure is further abbreviated by the term ‘full-information’.

#### Child psychopathology

Child psychopathology was rated by the children’s mothers using the German version of the Child Behavior Checklist for the Preschool Age (CBCL/1.5–5; [24,25]). The CBCL scales are widely accepted instruments for assessing behavioral and emotional symptoms in children of different ages, and they have proven reliability and validity [26]. The CBCL/1.5–5 consists of 100 items that are rated by parents on a 3-point-scale, and the Total Problems raw score serves as a measure for child psychopathology.

#### Maternal psychopathology

The self-report Symptom Checklist 90 Items-Revised (SCL 90-R; [27,28]) consists of 90 items (5-point scale: 1 = “no problem” to 5 = “very serious”) that cover a broad range of psychological and psychosomatic symptoms. The questionnaire measures one global factor that indicates general symptom stress, which is best represented by the Global Severity Index (GSI).

### Statistical analysis

The first step to analyze the reliability of video ratings was to compare their mean scores by a paired *t*-test. Second, the correlation between both video ratings was examined by a Pearson correlation. This first set of analyses was completed to determine if the video PIR-GAS ratings were interchangeable. Subsequently, both video ratings were combined by computing their mean. The rationale to form one combined video PIR-GAS score was that the full-information ratings used in this study were also ‘combined’ ratings, as they were the result of a team rating by a group of experts*.* Consequently, the combined video PIR-GAS score allowed for a fair comparison to the full-information ratings. Additionally, the combined video PIR-GAS score reduced the error variance that can be expected from single video ratings. We then compared the PIR-GAS combined video score with the full-information score by paired t-tests and Pearson correlations. Finally, the combined video and full-information ratings were validated by their correlation with the CBCL/1.5–5 Total Problem score and the GSI (from the SCL-90-R). All analyses with data from admission were replicated with data from discharge. Data were analyzed using SPSS Statistics 21.0 for Windows. Across all scales and measurement occasions, we achieved a rate of valid data of 83.54%. Despite this good result, single missing data points may imply a loss of data. We applied the SPSS 21 standard procedure for single imputation.

## Results

### Agreement between video ratings

The mean differences between the PIR-GAS ratings of the two video coders were not statistically significant (t_df=47_ = 1.838, n. s.; see Table 4). This result was replicated with data from discharge and again, the differences were not statistically significant (t_df=47_ = −0.252, n. s.).

**Table 4:** PIR-GAS ratings from two raters (1,2) on the basis of a 10-minute mother-child-interaction video compared to a group rating on basis of full clinical information at admission and discharge and supplementary Pearson correlations for interrater reliability and to external criteria (CBCL1.5-5; SCL-90-R GSI)

					**Video**		**Full-Info**
				***Coder 1***	***Coder 2***	***Coder 1,2***	***Clinical consensus rating***
	Mean (SD)	Admission		46.04^a^ (15.40)	41.67^a^ (19.92)	44.58 ^c^ (16.27)	36.29 ^c^ (13.71)
	Mean (SD)	Discharge		48.89 ^b^ (13.69)	50.28 ^b^ (19.20)	49.58 ^d^ (14.00)	47.22 ^d^ (14.33)
Interrater	Corr (p)	Admission		.570 (0.001)			
reliability	Corr (p)	Discharge	*Coder 2*	.509 (0.001)			
	Corr (p)	Admission					.057 (n. s.)
	Corr (p)	Discharge	*Coder 1,2*				.050 (n. s.)
	Corr (p)	Admission				.155 (n. s.)	-.056, (n. s.)
	Corr (p)	Discharge	*CBCL Tot*			.484 (0.001)	-.077 (n. s.)
Validity							
	Corr (p)	Admission				-.098 (n. s.)	.183 (n. s.)
	Corr (p)	Discharge	*SCL GSI*			-.119 (n. s.)	-.187 (n. s.)

^a,b,d^ Mean score difference not significant.

^c^ Significant mean score difference (p < .05) are indicated by the same letter.

*Coder 1,2* = a combined rating from Coder 1 and Coder 2.

CBCL Tot = Total Problem score from the CBCL/1.5-5.

SCL GSI = Global severity index from the SCL-90-R.

Furthermore, the video ratings were correlated significantly at admission (see Table 4). This result was replicated with data from discharge. For all subsequent analyses, we built a “video combined score” (Coder 1,2 in Table 4) using the mean of both single ratings to analyze differences and similarities with the full-information ratings.

### Agreement between video and full-information ratings

In t-tests for paired samples, the combined video rating and the full-information rating differed significantly from each other (t_df=47_ = 2.231, p = 0.031, see Table 4). The video ratings indicated a better relationship between mother and child at admission than did the full-information ratings, but this result was not replicated at discharge (t_df=47_ = 0.524, n. s.). The Pearson’s correlation between video and full-information ratings was very low and not significant. This result means that video and full-information coders gave differing ratings for the mother-child relationship. This finding was replicated at discharge.

### Validity of video and full-information PIR-GAS ratings

Finally, we present associations between the full-information and video PIR-GAS ratings, and external criteria (see Table 4). At admission, the combined video ratings showed no significant correlation with child psychopathology using the CBCL Total Problem score, but at discharge this correlation was significant. In terms of maternal psychopathology, we did not observe any significant correlation with the combined video rating at admission or at discharge. The full-information ratings were also not significantly correlated with child or maternal psychopathology at admission or discharge. In summary, we observed only one significant correlation out of the eight that we tested between the full-information and video PIR-GAS ratings and the two external criteria at admission and discharge.

## Discussion

### Conditions of PIR-GAS ratings for reliability and validity

A description and comparison of the ratings between the video ratings (paired *t*-test on mean score differences and correlations) suggests that both coders assessed approximately the same content and offered similar information about certain aspects of the mother-child relationship. This finding was interpreted as an aspect of the reliability of video ratings and allowed for combining both video ratings into one rating to compare them to the full-information ratings. The assessment procedure to conduct a PIR-GAS rating on a 10-minute interaction sample seems to allow a reliable, but not necessarily valid information about the mother-child-relationship quality. Therefore further analyses investigated the concordance to the full-information assessment procedure. Our results show, that the video coders rated the quality of the mother-child relationship considerably higher than the clinical staff did. A number of reasons may be responsible for these differences and will be discussed in detail next.

First, the ratings of video coders were based on a much smaller behavior sample compared to the full-information ratings. It is likely that a smaller sample of observations may lead to the impression of a higher quality of parent–child relationship, as some indicators of a dysfunctional relationship may occur too infrequently to be observed within a 10-minute interaction sample (e.g., arguing, shouting, or spanking). Second, the coders (doctoral candidates and experienced clinicians) may rely on different thresholds to rate a relationship as ‘disturbed’, which may be caused by different reference norms and unequal knowledge about clinical aspects of the infant-parent relationship. However, uncertainties exist not only for the 10-minute sample of interaction but also for the full-information rating. For example, it is unclear how well a clinician is able to integrate a large amount of potentially contradictory information, and the manual does not provide guidelines for how to process heterogeneous information, e.g., knowledge about child and familial circumstances. Finally clinicians might emphasize the pathology at admission to underline the need for treatment. This “bias” may also represent a self serving response set.

All of the aforementioned potential differences between video and full-information ratings may explain the low and insignificant correlation between both procedures. Therefore, in addition to the threshold problematic, the most important result of our study was that video and full-information ratings were not comparable. All aforementioned results were replicated with the data from discharge, except for one insignificant mean score difference. Further analyses focused on aspects of validity that examine the association of PIR-GAS ratings with known measures of child and maternal psychopathology. We only observed one significant association out of eight between the PIR-GAS ratings for the full-information and video ratings, and the measures of child or parental psychopathology at admission and discharge. These findings were somewhat unexpected, especially with regards to the validity of full-information ratings. Potential reasons are discussed in the following analysis of the PIR-GAS manual. We mentioned that our study design was primarily motivated by the paper of Aoki [19], where a PIR-GAS rating was based on a 10-minute video interaction sample by ‘blinded coders’, and showed predictive value to external criteria. We do not invalidate these findings with our study, but we questioned the equivalence of a 10-minute rating to a ‘full-information’ condition and did not find evidence that both measures can be used interchangeably. This issue was more closely addressed by the study of Salomonsson et al. [20], who reported a high intraclass interrater reliability. However, their external rating was not blinded with respect to admission or discharge assessment, which may affect the reported intraclass correlation. Moreover, the sample in Salomonsson et al. [20] was not comparable to ours, as their PIR-GAS mean scores considerably differed to mean score reported in our sample. Therefore, the results cannot be directly compared with each other.

### Analysis of the manual

The current status of instructions in the DC:0-3/DC:0-3R manual for how to conduct a PIR-GAS rating represent a theoretically desirable maximum. However, the studies that have already been conducted show that this desirable maximum is difficult to achieve in practical contexts and is even more difficult to achieve in a research setting. Therefore, we examine whether this maximum could be reduced to a practical minimum that would be desirable for research studies. For example, the manual states that clinical information from multiple sources, multiple observations, multiple methods, and multiple aspects should be integrated by an experienced and skilled clinician. Although the manual recommends the integration of all available information, and explicitly endorses taking parental distress into account ([2], p. 42), we suppose that a main intention of the DC:0-3R was to establish the PIR-GAS rating on Axis II as a new measure with its own incremental validity. As such, it should be independent from known measures (e.g., of child or parental distress) and should represent something new. In fact, we found that child and parental distress did not influence the PIR-GAS rating by full-information ratings. Consequently, our results point to the independence of the clinicians’ PIR-GAS judgments from other information, which is desirable from a methodological perspective.

We have identified several aspects to improve the DC:0-3/DC:0-3R with respect to conducting a PIR-GAS rating. Currently, a PIR-GAS rating can be conducted under very different circumstances according to the treatment/research settings and purpose. This idea renders the PIR-GAS ratings difficult to compare, irrespective of the individual degree of fulfillment of manual instructions. However, we see opportunities for further standardizations, for example, involving ‘relationship-relevant’ contents and recommended settings to observe the behavior of interest. Furthermore, it seems possible to define a set of criteria, which are already mentioned in the behavior anchored PIR-GAS levels, and a related coding scheme to increase agreement between different observers.

Aside from these aspects, it remains unknown whether further clinical information should be integrated into the final PIR-GAS rating. First, the necessary amount and quality of clinical information has not been sufficiently specified. Second, it is unclear how to integrate all of the available information. Finally, if additional clinical information (e.g., child and parental distress, maternal sensitivity, etc.) is integrated into the PIR-GAS rating, this clinical information cannot be used as external validity criteria of a PIR-GAS rating. Consequently, in contrast to the wording of the manual, the multiple facets of clinical information should not all be included in the relationship rating.

### Confounding of a classification system and its measurement tools

A classification system represents a framework for the interpretation of clinical observations, and for example, DSM and ICD provide explicit criteria to be fulfilled. A second characteristic of a nosological system is that it does not provide explicit measures to assess these criteria because this is a technical issue, and researchers can generally develop new measures on their own. These measures are in competition with each other and can be an issue of discussion without directly affecting the classification system in itself. Such a conceptual architecture implies an approach of permanently developing and improving measurement instruments. Unfortunately, the DC:0-3R, with the PIR-GAS directly included in AXIS II, confounds the level of classification with the level of assessment, which may lead to certain methodological problems. Specifically, when both levels are confounded, there are no external criteria left for empirical validation and evaluation of the classification system. Another problem arises with the theoretical background of the issue of the mother-child relationship. This core concept has not yet been sufficiently described, and a great number of similar concepts and terms exist in the literature (see below).

### Limitations

Our study design compared two procedures: ten-minute video coding and a group of clinician which base their rating on a maximum of clinical information. Actually, we can not say if the characteristics of the rater or the setting have lead to the low agreement. Therefore it is important to underline, that the observed low interrater-agreement between coders and clinicians is limited to the investigated condition. Coders and clinicians may achieve a much higher agreement if both ratings are based on comparable clinical information. Actually, we do not know how much information is necessary to give a reliable and valid estimation about the parent–child-relationship (see below).

Our results are also limited by the characteristic of the sample. In our sample, 56.3% of all mother-child dyads showed ‘disordered’ mother-child relationships at admission to therapy, according to Table 1 (PIR-GAS < 40) based on full-information ratings. This base rate was comparable to other psychiatric samples (see [8] with 52.4%; [9] with 52%; [18] with 40.5%). Moreover, the observed base rate represented a statistically desirable distribution of the quality of relationships, which allowed for describing the inter-rater reliability of coders. The interpretation of this study is limited by the small number of observers and the degree of standardization of videotaped mother-child interaction. Our video records showed situations of free mother-child interaction (mostly free-play situations), and results may differ from any high-structured or otherwise standardized setting. Upcoming experimental studies should focus on aspects of differences between observers (especially experience with children), observed material (duration and contents of the behavioral sample) and rating criteria (depending on the definition of parent–child relationship). Only a controlled variation of these factors will lead to more insight and might help to establish a standardized assessment of the quality of the parent–child relationship.

### Further research

The most important issue of upcoming research activities may be to clearly define the theoretical background of the relationship concept and its measures, in order to define a distinct and new concept and to develop measures with own incremental validity. Among the concurring terms which describe the parent–child relationship and are currently discussed in the literature, are for example maternal supportive presence, mother limit-setting, mother intrusiveness, mother-child joint positive affect, child withdrawal, dyadic joint negative state [9]; behavioral quality of the interaction, affective tone and psychological involvement [22]; involvement, positivity, hostility, intrusiveness, discipline [29]; emotional availability [30]; and tone of voice, parental affect, parents’ expressed attitudes toward the child, behavioral involvement, connectedness, mirroring, and joint attention [31].

Furthermore, what is viewed as a successful parent–child-interaction varies considerably depending on cultural background [32]. For this reason, Christensen and colleagues [33] have adjusted the guidelines of the Cultural Case Formulation from Appendix 1 of DSM-IV to meet the particular demands of assessing the early parent–child relationship. The pace of globalization suggests that this aspect may need to be considered when further revisions of the PIR-GAS are undertaken.

## Conclusions

The results of our study suggest that PIR-GAS ratings based on extensive clinical information and ratings based on a ten-minute interaction observation are not interchangeable, and that the validity of a PIR-GAS rating is somewhat questionable. We conclude that a higher degree of standardization of the assessment procedure should increase the reliability of the PIR-GAS, and that a more thorough theoretical foundation of the manual should increase its validity. We hope, that our study points to the necessity to find the optimum balance between time requirement and personal costs to achieve satisfying reliability and validity. Looking for an economical assessment of the parent–child-relationship may strengthen research activities in this field.

## Endnotes

^a^ For simplification, from now on, the term *DC:0-3/DC:0-3R* will be used to refer to both classification systems. If necessary, the version of focus will be specified.

## Competing interests

The authors declare that they have no competing interests.

## Authors‘ contributions

JM, SA , TB, TF and CP planned and supervised the study together. HF, KS and OS carried out the data collection and provided preliminary analyses. JM and SA conducted the final statistical analyses and interpretations. JM, SA and CP drafted the manuscript. All authors read and approved the final manuscript.

## References

[B1] Zero To Three/National Center for Infants Diagnostic classification of mental health and developmental disorders of infancy and early childhood: DC:0–3 1994 Washington, DC: Zero To Three

[B2] Zero To Three/National Center for Infants Diagnostic classification of mental health and developmental disorders of infancy and early childhood: DC: 0-3R 2005 Washington, DC: Zero To Three

[B3] Postert C Averbeck-Holocher M Beyer T Müller J Fürniss T Five systems of psychiatric classification for preschool children: do differences in validity, usefulness and reliability make for competitive or complimentary constellations? Child Psychiat Hum D 2009 40 25 41 10.1007/s10578-008-0113-x 18704679

[B4] Equit M Paulus F Fuhrmann Niemczyk J Von Gontard A Comparison of ICD-10 and DC: 0-3R diagnoses in infants, toddlers and preschoolers Child Psychiat Hum D 2011 42 623 633 10.1007/s10578-011-0237-2 21667157

[B5] DelCarmen-Wiggins R Carter A Handbook of infant, toddler, and preschool mental health assessment 2004 Oxford: Oxford University Press

[B6] Olson SL Bates JE Sandy JM Lanthier R Early development precursors of externalizing behavior in middle childhood and adolescence J Abnorm Child Psych 2000 28 119 133 10.1023/A:1005166629744 10834765

[B7] Shaw DS Owens EB Giovannelli J Winslow EB Infant and toddler pathways leading to early externalizing disorders J Am Acad Child Psy 2001 40 36 43 10.1097/00004583-200101000-00014 11195559

[B8] Minde K Tidmarsh L The changing practices of an infant psychiatry program: the McGill experience Infant Ment Health J 1997 18 135 144 10.1002/(SICI)1097-0355(199722)18:2<135::AID-IMHJ3>3.0.CO;2-O

[B9] Keren M Feldman R Tyano S A five-year Israeli experience with the DC:0–3 classification system Infant Ment Health J 2003 24 3337 3348

[B10] Skovgaard AM Houmann T Christiansen E Landorph S Jørgensen T CCC 2000 Study Team The prevalence of mental health problems in children 1½ years of age – the Copenhagen child cohort 2000 J Child Psychol Psyc 2007 48 62 70 10.1111/j.1469-7610.2006.01659.x 17244271

[B11] Thomas JM Clark R Disruptive behavior in the in the very young child: diagnostic classification 0–3 guides identification of risk factors and relational interventions Infant Ment Health J 1998 19 229 244 10.1002/(SICI)1097-0355(199822)19:2<229::AID-IMHJ10>3.0.CO;2-#

[B12] Donenberg G Baker B The impact of young children with externalizing behaviors on their families J Abnorm Child Psych 1993 21 179 198 10.1007/BF00911315 8491931

[B13] Emde RN Wise BK The cup is half full: initial clinical trials of DC: 0–3 and a recommendation for revision Infant Ment Health J 2003 24 437 446 10.1002/imhj.10067

[B14] Skovgaard AM Houmann T Christiansen E Andreasen AH The reliability of the ICD-10 and the DC 0–3 in an epidemiological sample of children 1½ years of age Infant Ment Health J 2005 26 470 480 10.1002/imhj.20065 28682496

[B15] Cantwell DP Classification of child and adolescent psychopathology J Child Psychol Psyc 1996 37 3 12 10.1111/j.1469-7610.1996.tb01377.x 8655656

[B16] Dunitz-Scheer M Scheer PJ Kvas E Macari S Psychiatric diagnoses in infancy: a comparison Infant Ment Health J 1996 17 12 24 10.1002/(SICI)1097-0355(199621)17:1<12::AID-IMHJ2>3.0.CO;2-3

[B17] Frankel KA Boyum LA Harmon RJ Diagnoses and presenting symptoms in an infant psychiatric clinic: a comparison of two diagnostic systems J Am Acad Child Psy 2004 43 578 587 10.1097/00004583-200405000-00011 15100564

[B18] Guedeney N Guedeney A Rabouam C Mintz AS Danon G Huet M Jacquemain F The zero-to-three diagnostic classification: a contribution to the validation of this classification from a sample of 85 under-threes Infant Ment Health J 2003 24 313 336 10.1002/imhj.10059

[B19] Aoki Y Zeanah CH Scott Heller S Bakshi S Parent-infant relationship Global assessment scale: a study of its predictive validity Psychiat Clin Neuros 2002 56 493 497 10.1046/j.1440-1819.2002.01044.x 12193237

[B20] Salomonsson B Sandell R A randomized controlled trial of mother–infant psychoanalytic treatment: I. outcomes on self-report questionnaires and external ratings Infant Ment Health J 2011 32 207 231 10.1002/imhj.20291 26908296

[B21] Salomonsson B Sandell R A randomized controlled trial of mother–infant psychoanalytic treatment: II. predictive and moderating influences of qualitative patient factors Infant Ment Health J 2011 32 377 404 10.1002/imhj.20302 26904966

[B22] Boris NW Zeanah CH Larrieu JA Scheeringa MS Heller SS Attachment disorders in infancy and early childhood: a preliminary investigation of diagnostic criteria Am J Psychiatry 1998 155 295 297 9464217 10.1176/ajp.155.2.295

[B23] von Hofacker N Papoušek M Disorders of excessive crying, feeding, and sleeping: the Munich interdisciplinary research and intervention program Inf Ment Health J 1998 19 180 201 10.1002/(SICI)1097-0355(199822)19:2<180::AID-IMHJ7>3.0.CO;2-S

[B24] Achenbach TM Rescorla LA Manual for the ASEBA preschool forms and profiles 2000 Burlington, VT: University of Vermont Department of Psychiatry

[B25] Arbeitsgruppe Deutsche Child Behavior Checklist Elternfragebogen für Klein- und Vorschulkinder (CBCL/1,5-5) [Questionary for parents of toddlers und preschool children (CBCL/1,5-5)] 2002 Arbeitsgruppe Kinder-, Jugend- und Familiendiagnostik: Köln

[B26] Rescorla LA Assessment of young children using the Achenbach system of empirically based assessment (ASEBA) Ment Retard Dev D R 2005 11 226 237 10.1002/mrdd.20071 16161094

[B27] Derogatis LR SCL-90-R, administration, scoring and procedures manual-II for the R(evised) version and other instruments of the psychopathology rating scale series 1992 Townson: Clinical Psychometric Research Inc.

[B28] Franke GH SCL-90-R - Symptom-Checkliste von L.R. Derogatis 2002 Beltz Test GmbH: Weinheim

[B29] Wilson S Durbin CE The laboratory parenting assessment battery: development and preliminary validation of an observational parenting rating system Psychol Assessment 2012 24 823 832 10.1037/a0028352 22545699

[B30] Biringen Z Easterbrooks MA Emotional availability: concept, research, and window on developmental psychopathology Dev Psychopathol 2012 24 1 8 10.1017/S0954579411000617 22292989

[B31] Clark R The parent–child early relational assessment: instrument and manual 1985 Madison, WI: University of Wisconsin Medical School, Department of Psychiatry

[B32] Carter AS Briggs-Gowan MJ Davis NO Assessment of young children’s social-emotional development and psychopathology: Recent advances and recommendations for practice J Child Psychol Psyc 2004 45 109 134 10.1046/j.0021-9630.2003.00316.x 14959805

[B33] Christensen M Emde Fleming C DelCarmen Wiggins R, Carter A Cultural Perspectives for assessing infants and young children Handbook of infant, toddler and preschool mental health assessment 2004 Oxford: Oxford University Press 7 23

